# Universal Genotyping in Tuberculosis Control Program, New York City, 2001–2003

**DOI:** 10.3201/eid1205.050446

**Published:** 2006-05

**Authors:** Carla M. Clark, Cynthia R. Driver, Sonal S. Munsiff, Jeffrey R. Driscoll, Barry N. Kreiswirth, Benyang Zhao, Adeleh Ebrahimzadeh, Max Salfinger, Amy S. Piatek, Jalaa' Abdelwahab, the New York City Molecular Epidemiology Working Group

**Affiliations:** * New York City Department of Health and Mental Hygiene, New York, New York, USA;; † Centers for Disease Control and Prevention, Atlanta, Georgia, USA;; ‡ New York State Department of Health's Wadsworth Center, Albany, New York, USA;; § Public Health Research Institute, Newark, New Jersey, USA;; ¶ Public Health Laboratories, New York, New York, USA

**Keywords:** Genotyping, tuberculosis, epidemiology, transmission, perspective

## Abstract

In 2001, New York City implemented genotyping to its tuberculosis (TB) control activities by using IS*6110* restriction fragment length polymorphism (RFLP) and spoligotyping to type isolates from culture-positive TB patients. Results are used to identify previously unknown links among genotypically clustered patients, unidentified sites of transmission, and potential false-positive cultures. From 2001 to 2003, spoligotype and IS*6110*-based RFLP results were obtained for 90.7% of eligible and 93.7% of submitted isolates. Fifty-nine (2.4%) of 2,437 patient isolates had false-positive culture results, and 205 genotype clusters were identified, with 2–81 cases per cluster. Cluster investigations yielded 57 additional links and 17 additional sites of transmission. Four additional TB cases were identified as a result of case finding initiated through cluster investigations. Length of unnecessary treatment decreased among patients with false-positive cultures.

## Abstract 2

Real-time universal genotyping decreased unnecessary treatment.

Since the early 1990s, selective tuberculosis (TB) genotyping has been used in New York City for outbreak investigations, to identify isolates resistant to at least isoniazid and rifampin (multidrug-resistant TB), and in special studies. TB genotyping was essential to investigate and confirm transmission in a number of settings and to confirm or exclude laboratory contamination (*1**–**8*). A number of programs demonstrated the utility of universal genotyping, which influenced the development of this service in New York City (*9**–**16*). In 2001, the New York City Bureau of Tuberculosis Control began genotyping isolates for every new TB case with spoligotyping and IS*6110*-based restriction fragment length polymorphism (RFLP) to improve the efficiency of TB control. Two laboratories with extensive genotyping experience were selected through a competitive bidding process. Both were participating laboratories in the National Tuberculosis Genotyping and Surveillance Network and had performed genotyping for selected cases in New York City since the early 1990s (*6**,**17*).

The objectives of universal TB genotyping were to more rapidly and efficiently 1) determine the extent and dynamics of ongoing transmission to focus program interventions for specific areas and populations; 2) assess TB transmission in outbreaks to refine contact investigations; 3) identify nosocomial transmission not identified by conventional methods; and 4) identify false-positive cultures so that clinicians could be notified of diagnostic errors quickly and prevent unnecessary TB treatment. We describe the elements and activities required to develop and implement real-time universal genotyping in a large urban TB control program.

## Identifying and Obtaining Isolates for Genotyping

Implementation of universal genotyping in New York City consisted, briefly, of 1) requiring submission of the initial positive isolate, reinforced by health code amendment (*18**,**19*); 2) advising all relevant laboratories and providers of new requirements; 3) modifying laboratory submission forms; 4) establishing a specimen transport system; and 5) tracking and reviewing all submissions. In addition, protocols were developed for surveillance of genotype results and false-positive culture investigations, existing patient interview forms were modified, new databases were created, and program staff were informed through special trainings and newsletters. The New York City Department of Health and Mental Hygiene Institutional Review Board and the associate director for Science of the National Center for HIV, STD, and TB Prevention, Centers for Disease Control and Prevention, reviewed the protocols, procedures, and modified data forms and determined that the genotyping service was not human subjects research since it would become a routine program activity.

## Laboratory Procedures

An additional full-time staff person was hired by the Bureau of Tuberculosis Control to coordinate genotyping services. Spoligotyping and RFLP, respectively, were performed by the New York State Department of Health's Wadsworth Center in Albany, New York, and the Public Health Research Institute in Newark, New Jersey. This combination of genotyping methods is sensitive and specific for determining matching genotypes (*20**–**24*).

Isolates of *Mycobacterium tuberculosis* complex submitted to the public health laboratory from clinical laboratories were received on solid or liquid media and were stored at 4°C. Liquid media were prepared (10% glycerol in Dubos Davis broth with Tween and albumin), 1 mL of liquid culture was injected and incubated for 3 days at 37°C and checked visually for growth. Four freeze vials (1 for spoligotyping and 3 for archiving) and 1 Lowenstein-Jensen slant were injected. Mycobacteria in the tubes for spoligotyping were heat-killed at 80°C for 1 hour and mailed in biohazard containers to the Wadsworth Center. Once appropriate growth was obtained on the Lowenstein-Jensen slants, they were sent in a biohazard container to the Public Health Research Institute for IS*6110* RFLP analysis. Packages were mailed on a weekly or biweekly basis, depending on the number of isolates received. Spoligotype analysis was performed at the Wadsworth Center and given descriptive nomenclature according to a standard method (*25**–**27*). DNA analysis based on IS*6110* Southern blot hybridization was performed at the Public Health Research Institute with previously described methods (*28**,**29*). To ensure good communication, a working group of all partners in the genotyping service was formed. Regular telephone conferences were conducted to address issues such as quality and shipping of isolates and submission time for genotyping.

## Creation of TB Genotyping Databases

Implementing universal genotyping also required developing a comprehensive database to monitor and manage information on specimen collection, shipment, and genotyping, as well as epidemiologic information gathered on each clustered patient. A relational database was created by New York City TB control staff in Microsoft Access (Microsoft Corporation, Redmond, WA, USA) that included 1) genotyping results for isolates identified after January 1, 2001; 2) specimen-tracking information such as date of receipt at the public health laboratory, shipment and reporting dates from each genotyping laboratory, and false-positive culture investigation results; 3) clustered patient information, such as location where each patient spent time during the potential infectious period, locations where TB could have been acquired in the 5 years before diagnosis, cluster characteristics, links between patients, and potential transmission sites; and 4) results of genotyping performed from 1990 to 2000 as part of the selective genotyping activities (*3**,**5**,**6**,**8**,**17*). Queries of the database were developed to identify cases with identical RFLP and spoligotype results for "real-time" cluster investigation and investigations of false-positive cultures. Quality assurance exercises to test reliability of results were developed and kept in the database. Queries are performed monthly to identify cases for which an isolate was not submitted to the public health laboratory. In such cases, Bureau of Tuberculosis Control staff sends reminder letters and makes phone calls to ensure that these isolates are received.

## Application of Universal Genotyping Data

### Investigation of False-positive Culture Results

A false-positive TB culture is defined as a positive TB culture that is not the result of culture-positive disease in a patient but instead may be due to 1) laboratory cross-contamination during specimen processing; 2) errors in collection or labeling, either on the patient ward or in the laboratory; or 3) contamination of clinical devices, for example, contamination of a bronchoscope during specimen collection. The primary goal of investigations of false-positive cultures is to discontinue unnecessary treatment in patients found to have false-positive TB cultures. Before universal genotyping, suspected false-positive cultures were investigated in 1 of 3 ways: 1) monthly review of patients with a single positive culture; 2) request from Bureau of Tuberculosis Control staff, including case managers, department of health physicians, and epidemiologists; and 3) requests by outside providers and laboratories to investigate cultures not consistent with the patient's clinical picture. With universal genotyping, an investigation can also be initiated when cases have matching genotypes and have been processed within 2 working days of each other at the same facility. For these investigations, genotype information, specimen processing, and other information (e.g., patients hospitalized on the same floor) are reviewed, and suspected false-positive cultures are determined to be confirmed, unlikely, or inconclusive. Treating physicians and clinical staff in the program are notified of the outcome of investigations of false-positive cultures so patient evaluation can be evaluated further and a decision can be made on whether continued treatment is indicated.

### Genotype Cluster Investigations

A cluster investigation aims to uncover epidemiologic links between members of a genotype cluster through systematic review of patient records and re-interviews, if needed. We consider real-time investigation of clusters to occur when the cluster investigation components (i.e., record review and re-interview) take place close to the time the most recent case in the cluster is identified. We defined a genotype cluster as >2 cases identified from 2001 to 2003 that had isolates with identical IS*6110*-based RFLP banding pattern and spoligotype, regardless of the number of IS*6110* copies. Patients with a definite epidemiologic link include those who have named each other as contacts, have a contact in common without naming each other as contacts, or have reported a common date range at the same location (e.g., residence, hospital, prison, workplace, single-room-occupancy hotel [any supervised publicly or privately operated facility designed to provide temporary living accommodations], or shelter). The common date range includes the potentially infectious period (i.e., 3 months before start of treatment) for at least 1 patient. Patients with a probable link have spent time at the same location (as above) during the same time frame, exclusive of the infectious period of the patients, without naming each other as contacts. Possible links exist among patients who have a similar social network or have spent time in the same area (no specific location), without naming each other as contacts.

When definite epidemiologic links are found among cluster members through the review of the TB case registry and patient records, information is recorded in the database on the nature of this relationship. If transmission at specific locations is shown, additional contacts are tested at these locations. If no such links exist, an epidemiologist reviews the cases and conducts in-depth patient re-interview to attempt to identify links and previously unidentified locations of transmission. A standard questionnaire is used to re-interview clustered patients. In addition, other registries such as the Department of Homeless Services are searched by cross-matching with the database each quarter to identify other possible exposure locations.

## Performance Indicators

Performance indicators are used to evaluate procedures with respect to timely shipping of isolates and reporting of genotyping results and to assess the reliability of genotyping results. Submission time is calculated for clinical laboratories that process samples from New York City TB patients as the time between the date a positive culture is collected and the date the isolate is received at the public health laboratory. Submission time for genotyping is the time between the date the isolate is received at the public health laboratory and the date the isolate is sent for genotyping. Reporting time for the genotyping laboratories is the time between the date the specimen is received at the genotyping laboratory and the date the spoligotype or RFLP is reported to the Bureau of Tuberculosis Control.

The time to completion of investigations of false-positive cultures is defined as the time from specimen collection to investigation completion. The goal is to complete investigations within 90 days of collecting the first positive culture. Time to completion of a cluster investigation is calculated from the date a cluster is identified and an investigation is initiated until a decision is made regarding links between cases in the cluster; the goal is to complete these investigations within 21 days. Because clusters are dynamic, a new investigation is started when an additional case with that particular strain is identified.

Quality assurance exercises to assess the reliability of genotyping results are performed every 6 months. Ten percent of isolates genotyped in the previous 6 months are randomly selected by Bureau of Tuberculosis Control and sent for blinded retyping. Each laboratory repeats genotyping and sends the results to the bureau for comparison with previously reported results. Discrepant results are reviewed and discussed in the working group, and another isolate is requested from the initial processing laboratory to verify results.

## Outcomes

**Figure A1:**
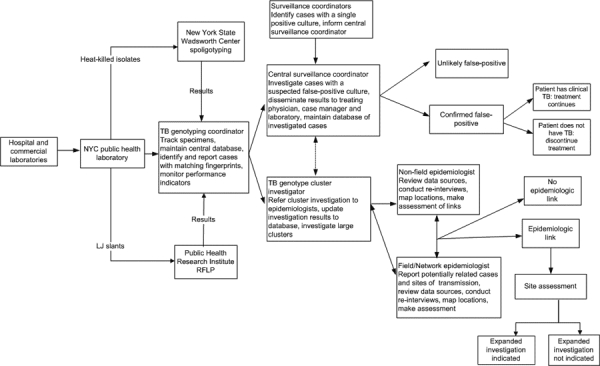
Summary of tuberculosis (TB) genotyping activities, New York City, 2001–2003. NYC, New York City; LJ, Lowenstein-Jensen; RFLP, restriction fragment length polymorphism.

The genotyping services process is summarized in the Figure A1. The number of eligible isolates by year is shown in the Table. As of March 2004, isolates for 2,600 (96.8%) of 2,685 patients with a diagnosis of culture-positive TB from January 1, 2001, to December 31, 2003, were submitted. Of 85 patient isolates not submitted to the public health laboratory, 78.8% were processed at commercial laboratories, mostly outside of New York City. For patient isolates with incomplete genotyping (n = 163), RFLP could not be performed because of inadequate growth or overgrowth with other mycobacteria or fungi. Spoligotype and RFLP results were available for 2,437 (93.7%) of the 2,600 isolates submitted (90.7% of all culture-positive patients). The median days from specimen collection to reporting of spoligotype decreased from 84 days in 2001 to 53 days in 2003, and the reporting time for RFLP patterns decreased from 127 days in 2001 to 78 days in 2003 (Figure). Fifty-nine (2.4%) isolates were false-positive cultures; 37% of investigations of these false-positive cultures were initiated through matching genotyping results or a spoligotype suggestive of contamination with a laboratory TB strain. Outside requests initiated 8.5% of investigations; 24.0% were initiated from the single positive culture list, and 30.5% by request from staff within the Bureau of Tuberculosis Control. The median time to complete investigations of false-positive cultures decreased from 178 days in 2001 to 85 days in 2003. In 2003, patients with a false-positive culture were treated unnecessarily for a median of 7 days (range 0–145). This median number of days is considerably lower than that seen before universal genotyping; in 1999, patients identified by retrospective surveillance (i.e., the single-positive culture list) as having false-positive cultures completed a median of 7 months of treatment.

**Table:** Genotyping of isolates, New York City, 2001–2003*

Isolate characteristics	2001, no. (%)	2002, no. (%)	2003, no. (%)
Culture-positive	965 (100)	840 (100)	880 (100)
Culture received by PHL	928 (96.2)	808 (96.2)	864 (98.2)
Complete genotype (RFLP plus spoligotype)	883 (95.2)	772 (95.5)	782 (90.5)
Clustered genotypes	311 (36.3)†	262 (34.9)†	264 (34.2)†

*PHL, public health laboratory; RFLP, restriction fragment length polymorphism. †Excludes false-positive cultures.

**Figure:**
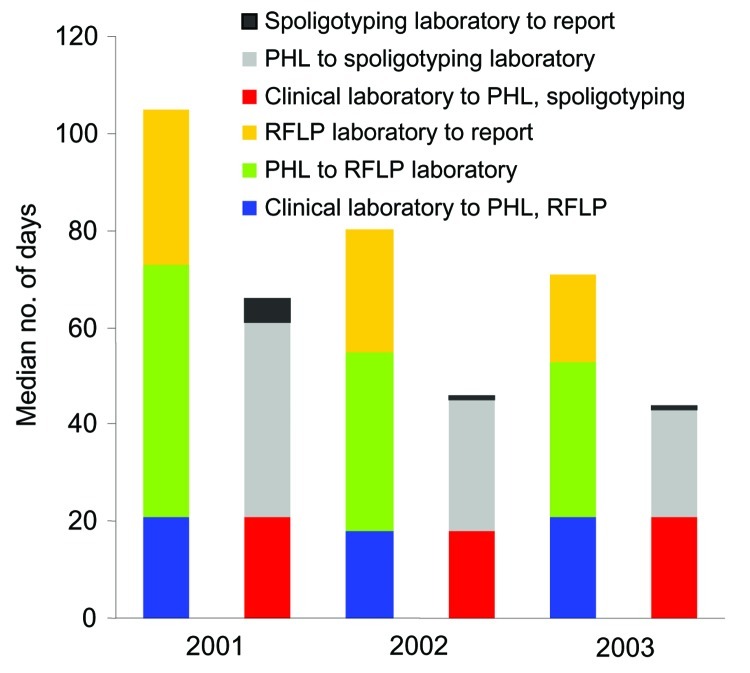
Median days for submission and turnaround time by laboratory, New York City, 2001–2003. PHL, public health laboratory; RFLP, restriction fragment length polymorphism.

Among 2,378 isolates with a complete genotype (true-positive cultures), 565 spoligotype patterns and 1,600 RFLP patterns were identified; 2,009 (84.5%) of 2,378 patient isolates clustered in 196 spoligotype clusters, and 1,002 (42.1%) of 2,378 patient isolates in 224 RFLP clusters. Eight hundred thirty-seven (35.2%) of the 2,378 isolates had RFLP and spoligotype patterns that matched >1 other isolate pattern; these isolate patterns were grouped into 205 genotype clusters ranging in size from 2 to 81 cases (mean 4 cases/cluster; median 2 cases/cluster). The percentage of clustered patient isolates remained stable during the 3-year period (χ^2^ for trend p = 0.3652). While most patient isolates had 9–13 copies of IS*6110* (median 11 copies, range 1–23), strains with a lower copy number (<6 bands) were more likely to be clustered. From 2001 to 2003, two large outbreaks occurred that involved strains of 1 and 3 IS*6110* copies. After these strains were excluded, the percentage clustered remained higher for patterns with lower numbers of IS*6110*.

A total of 278 (33.2%) of 837 clustered cases had epidemiologic links identified; of these, 105 (37.8%) had links established through traditional contact investigations. Genotype cluster investigations established links for the remaining 62%: 15% of the links were definite, 11% probable, and 36% possible. For 66.4% of clustered cases (556 cases), no epidemiologic links were identified. Time to completion of cluster investigations decreased from a median of 176 days in 2001 to 37 days in 2003. The delay in completing investigations at the beginning of the project was mostly due to staff vacancies. Cluster investigations uncovered 57 additional links among cases with matching genotypes and 17 additional sites of transmission. Links established through genotype cluster investigations led to 4 expanded contact investigations in congregate settings (2 in homeless shelters, 1 in a single-room-occupancy hotel, and 1 in a local grocery store). These investigations identified additional infected contacts and 4 additional TB patients at a homeless shelter. These sites are now monitored closely for additional patient isolates with these genotypes. Transmission between TB patients was ruled out in >5 site investigations because the genotypes were unrelated, avoiding more extensive case-finding efforts that are needed once transmission is seen.

Four quality assurance exercises were performed from 2001 to 2003 on 216 isolates. The result was 94.4% concordance for spoligotyping and 93.5% for RFLP. Of retyped spoligotype patterns that did not exactly match the original patterns, 50% differed by ±1 spacer, 8% differed in multiple successive spacers, and 42% differed for other reasons. Among retyped RFLP patterns, 57% differed from the original patterns because of the existence or absence of >1 bands, 36% differed because of pattern shifts, and 7% differed for other reasons.

## Discussion

We achieved real-time universal genotyping as part of routine TB control with capture and completion comparable to that seen by the National Tuberculosis Genotyping and Surveillance Network sites (*30*). High participation rates among clinical laboratories were essential to the completeness of genotyping. Timely submission of isolates from clinical laboratories and continuing decrease in submission time from the public health laboratory to the genotyping laboratories also facilitated efforts to achieve real-time investigation of false-positive cultures and clusters.

Implementation of TB genotyping in a large TB control program is complex. It requires TB control, epidemiology, and laboratory resources, and the costs are substantial. New York City contracts with genotyping laboratories carry an annual cost of nearly US $150,000 ($20,000 for spoligotyping and $125,000 for RFLP). In addition, 2 to 3 epidemiologists are allocated for database management and cluster investigation in New York City. Nonetheless, we have seen added value from universal genotyping. Additional sites of transmission were found on the basis of results of cluster investigations. Expanded investigations conducted at these sites identified additional patients and infected contacts who were subsequently treated for TB and latent TB infection. Genotyping information has also been useful by showing that TB cases clustered in place and time can have unrelated genotypes. For example, unrelated genotypes of >2 TB cases diagnosed in a setting with a high prevalence of TB infection may provide evidence that the cases did not occur as a result of transmission within that setting. Thus, more limited contact investigations of persons exposed to each of the patients can be performed instead of the more aggressive expanded contact investigation or case-finding activities that would be required if the isolates had matching genotypes. In addition, the efficiency of investigations of false-positive cultures increased as a result of universal genotyping, since a greater proportion of investigations initiated through genotyping matches yielded true false-positive culture results than investigations initiated through other methods. The amount of unnecessary treatment for these patients also decreased.

The higher rates of clustering seen in low copy-number isolates by RFLP alone support our decision to use 2 genotyping assays; this phenomenon has been reported previously (*11*). In addition, the rapid availability of spoligotype results allowed earlier initiation of investigations of both clusters and false-positive cultures than would have been possible with RFLP results alone. Particularly useful was close communication with the Wadsworth Center on interpretation of spoligotype matches for "rare" spoligotypes (seen less often than average for most spoligotypes in our database) and on prioritization of these isolates for investigation as clusters or false-positive cultures.

Since January 2004, mycobacterial interspersed repetitive unit and spoligotyping analyses are performed on all isolates as part of the Centers for Disease Control and Prevention's National Tuberculosis Genotyping Program. The availability of this additional assay will allow us to examine the extent to which MIRU further differentiates genotype clusters on the basis of RFLP and spoligotyping. MIRU may also reduce the time to obtain the genotype result and initiate a cluster investigation since it, like spoligotyping, requires few organisms and does not require live culture. Implementing the national genotyping service will also greatly reduce the financial costs for TB control jurisdictions interested in using genotyping to enhance their current program activities (*31*).

## References

[1] Valway SE, Richards SB, Kovacovich J, Greifinger RB, Crawford JT, Dooley SW. Outbreak of multi-drug-resistant tuberculosis in a New York State prison, 1991. Am J Epidemiol. 1994;140:113–22. 8023800 10.1093/oxfordjournals.aje.a117222

[2] Coronado VG, Beck-Sague CM, Hutton MD, Davis BJ, Nicholas P, Villareal C, Transmission of multidrug-resistant *Mycobacterium tuberculosis* among persons with human immunodeficiency virus infection in an urban hospital: epidemiologic and restriction fragment length polymorphism analysis. J Infect Dis. 1993;168:1052–5. 10.1093/infdis/168.4.1052 8104226

[3] Frieden TR, Sherman LF, Maw KL, Fujiwara PI, Crawford JT, Nivin B, A multi-institutional outbreak of highly drug resistant tuberculosis. JAMA. 1996;276:1229–35. 10.1001/jama.1996.03540150031027 8849750

[4] Alland D, Kalkut GE, Moss AR, McAdam RA, Hahn JA, Bosworth W, Transmission of tuberculosis in New York City. An analysis by DNA fingerprinting and conventional epidemiologic methods. N Engl J Med. 1994;330:1710–6. 10.1056/NEJM199406163302403 7993412

[5] Nivin B, Nicholas P, Gayer M, Frieden TR, Fujiwara PI. A continuing outbreak of multidrug-resistant tuberculosis, with transmission in a hospital nursery. Clin Infect Dis. 1998;26:303–7. 10.1086/516296 9502446

[6] Munsiff SS, Bassoff T, Nivin B, Li J, Sharma A, Bifani P, Molecular epidemiology of multidrug-resistant tuberculosis, New York City, 1995–1997. Emerg Infect Dis. 2002;8:1230–8. 12453347 10.3201/eid0811.020288 PMC2737807

[7] Frieden TR, Fujiwara PI, Washko RM, Hamburg MA. Tuberculosis in New York City—turning the tide. N Engl J Med. 1995;333:229–33. 10.1056/NEJM199507273330406 7791840

[8] Frieden TR, Woodley CL, Crawford JT, Lew D, Dooley SM. The molecular epidemiology of tuberculosis in New York City: the importance of nosocomial transmission and laboratory error. Tuber Lung Dis. 1996;77:407–13. 10.1016/S0962-8479(96)90112-4 8959143

[9] Castro KG, Jaffe HW. Rationale and methods for the National Tuberculosis Genotyping and Surveillance Network. Emerg Infect Dis. 2002;8:1188–91. 12453341 10.3201/eid0811.020408 PMC2738540

[10] Crawford JT, Braden CR, Schable BA, Onorato IM. National Tuberculosis Genotyping and Surveillance Network: design and methods. Emerg Infect Dis. 2002;8:1192–6. 12453342 10.3201/eid0811.020296 PMC2737808

[11] Cowan LS, Crawford JT. Genotype analysis of *Mycobacterium tuberculosis* isolates from a sentinel surveillance population. Emerg Infect Dis. 2002;8:1294–302. 12453359 10.3201/eid0811.020313 PMC2738546

[12] Small PM, Hopewell PC, Singh SP, Paz A, Parsonnet J, Ruston DC, The epidemiology of tuberculosis in San Francisco—a population-based study using conventional and molecular methods. N Engl J Med. 1994;330:1703–9. 10.1056/NEJM199406163302402 7910661

[13] Jasmer RM, Hahn JA, Small PM, Daley CL, Behr MA, Moss AR, A molecular epidemiologic analysis of tuberculosis trends in San Francisco, 1991–1997. Ann Intern Med. 1999;130:971–8. 10383367 10.7326/0003-4819-130-12-199906150-00004

[14] van Soolingen D, Borgdorff MW, de Haas PEW, Sebek MMGG, Veen J, Dessens M, Molecular epidemiology of tuberculosis in the Netherlands: a nationwide study from 1993 through 1997. J Infect Dis. 1999;180:726–36. 10.1086/314930 10438361

[15] van Deutekom H, Gerritsen JJJ, van Soolingen D, van Ameijden EJC, van Embden JDA, Coutinho RA. Molecular epidemiological approach to studying the transmission of tuberculosis in Amsterdam. Clin Infect Dis. 1997;25:1071–7. 10.1086/516072 9402360

[16] Borgdorff MW, Nagelkerke N, van Soolingen D, de Haas PE, Veen J, van Embden JD. Analysis of tuberculosis transmission between nationalities in the Netherlands in the period 1993–1995 using DNA fingerprinting. Am J Epidemiol. 1998;147:187–95. 9457010 10.1093/oxfordjournals.aje.a009433

[17] Fujiwara PI, Cook SV, Rutherford CM, Crawford JT, Glickman SE, Kreiswirth BN, A continuing survey of drug-resistant tuberculosis, New York, April 1994. Arch Intern Med. 1997;157:531–6. 10.1001/archinte.1997.00440260077012 9066457

[18] New York City Department of Health and Mental Hygiene, Bureau of Tuberculosis Control Annual Report. New York: The Department; 2001.

[19] New York City Public Health Code, Article 13, Section 13.05, 2000.

[20] Kremer K, van Soolingen D, Frothingham R, Haas WH, Hermans PWM, Martin C, Comparison of methods based on different molecular epidemiological markers for typing of *Mycobacterium tuberculosis* complex strains: interlaboratory study of discriminatory power and reproducibility. J Clin Microbiol. 1999;37:2607–18. 10405410 10.1128/jcm.37.8.2607-2618.1999 PMC85295

[21] Soini H, Pan X, Teeter L, Musser JM, Graviss EA. Transmission dynamics and molecular characterization of *Mycobacterium tuberculosis* isolates with low copy number of IS*6110.* J Clin Microbiol. 2001;39:217–21. 10.1128/JCM.39.1.217-221.2001 11136774 PMC87705

[22] Goyal M, Saunders NA, van Embden JDA, Young DB, Shaw RJ. Differentiation of *Mycobacterium tuberculosis* isolates by spoligotyping and IS*6110* restriction fragment length polymorphism. J Clin Microbiol. 1997;35:647–51. 9041405 10.1128/jcm.35.3.647-651.1997 PMC229643

[23] Bauer J, Andersen AB, Kremer K, Miörner H. Usefulness of spoligotyping to discriminate IS*6110* low-copy-number *Mycobacterium tuberculosis* complex strains cultured in Denmark. J Clin Microbiol. 1999;37:2602–6. 10405409 10.1128/jcm.37.8.2602-2606.1999 PMC85294

[24] Rasolofo-Razanamparany V, Ramarokoto H, Aurëgan G, Gicquel B, Chanteau S. A combination of two genetic markers is sufficient for restriction fragment length polymorphism typing of *Mycobacterium tuberculosis* complex in areas with a high incidence of tuberculosis. J Clin Microbiol. 2001;39:1530–5. 10.1128/JCM.39.4.1530-1535.2001 11283082 PMC87965

[25] Kamerbeek J, Schouls L, Kolk A, van Agterveld D, van Soolingen D, Kuijpers S, Simultaneous detection and strain differentiation of *Mycobacterium tuberculosis* for diagnosis and epidemiology. J Clin Microbiol. 1997;35:907–14. 9157152 10.1128/jcm.35.4.907-914.1997 PMC229700

[26] Driscoll JR, Bifani PJ, Mathema B, McGarry MA, Zickas GM, Kreiswirth BN, Spoligologos: a bioinformatic approach to displaying and analyzing *Mycobacterium tuberculosis* data. Emerg Infect Dis. 2002;8:1306–9. 12453361 10.3201/eid0811.020174 PMC2738554

[27] Dale JW, Brittain D, Cataldi AA, Cousins D, Crawford JT, Driscoll J, Spacer oligonucleotide typing of bacteria of the *Mycobacterium tuberculosis* complex: recommendations for standard nomenclature. Int J Tuberc Lung Dis. 2001;5:216–9. 11326819

[28] Kreiswirth BN, Moss AR. Genotyping multidrug-resistant *M. tuberculosis* in New York City. In: Rom WN, Garay SM, editors. Tuberculosis. Boston: Little, Brown, and Company, Inc; 1996. p. 199–209.

[29] van Embden JD, Cave MD, Crawford JT, Dale JW, Eisenach KD, Gicquel B, Strain identification of *Mycobacterium tuberculosis* by DNA fingerprinting: recommendations for a standardized methodology. J Clin Microbiol. 1993;31:406–9. 8381814 10.1128/jcm.31.2.406-409.1993 PMC262774

[30] Ellis BA, Crawford JT, Braden CR, McNabb SJN, Moore M, Kammerer S, Molecular epidemiology of tuberculosis in a sentinel surveillance population. Emerg Infect Dis. 2002;8:1197–209. 12453343 10.3201/eid0811.020403 PMC2738559

[31] National TB Controllers Association/Centers for Disease Control Advisory Group on Tuberculosis Genotyping. Guide to the application of genotyping to tuberculosis prevention and control. Atlanta: US Department of Health and Human Services; 2004.

